# Human Papillomavirus awareness and vaccine acceptability among men who have sex with men from mainland China

**DOI:** 10.1038/s41598-019-45258-0

**Published:** 2019-06-19

**Authors:** Xiangwei Li, Xuefang Cao, Zhen Li, Yu Yang, Mufei Li, Boxuan Feng, Henan Xin, Haoran Zhang, Lei Gao

**Affiliations:** 1 NHC Key Laboratory of Systems Biology of Pathogens, Institute of Pathogen Biology, Chinese Academy of Medical Sciences & Peking Union Medical College, Beijing, China; 2 Chaoyang Centers for Disease Control and Prevention, Beijing, China; 3 Department of Epidemiology and Biostatistics, School of Public Health, Peking University Health Science Centre, Beijing, China

**Keywords:** Epidemiology, Infection

## Abstract

Reductions in persistent HPV infection and related diseases occurrence have been proved among vaccinated males. However, little was known on awareness of HPV and the vaccine in males, especially in high-risk subgroups such as men who have sex with men (MSM), in China. A multicenter cross-sectional study was conducted in MSM from 10 selected cities in mainland China. HPV awareness and vaccination acceptability were investigated through interviews and questionnaires. In total, 3057 eligible participants aged 18 years older from 10 cities were investigated. Only 20.6% (629/3057) of them had ever heard of HPV and 4.8% (146/3057) had heard of HPV vaccine. Factors that potentially influence willingness for HPV vaccination were found to be safety of the vaccine (54.2%, 1656/3057) and severity of HPV infection (52.3%, 1599/3057). After education, 97.8% (2882/2946) of participants would like to pay for HPV vaccination, and only a minority of them (2.5%, 75/2946) would like to afford more than 2000 RMB. Our results showed that the awareness on HPV and the vaccine were quite poor among MSM in mainland China. To promote the application of HPV vaccination in male populations, appropriate information delivery and education on HPV infection and health should be enhanced as well as in females.

## Introduction

The human papillomavirus (HPV) anogenital infection is one of the common sexually transmitted infections around the world^1^. Persistent infection of high-risk HPV genotypes (including types 16, 18, 31, 33, 45, 52 and 58) can cause cancers, including cervical cancer, penile cancer, anal cancer, as well as a fraction of head and neck cancers. Whereas low-risk HPV genotypes (including types 6 and 11) are pathogens of anogenital warts^2–4^. Several published studies have demonstrated that men who have sex with men (MSM) were at higher risk of HPV/HIV co-infection than heterosexual males due to their more common risky sexual behaviors, such as having unprotected anal sex and multiple sexual partners^5–8^. This leads to an increased risk of anogenital cancer among HIV-positive MSM^9,10^. Approximately 85% of anal cancers and 50% of penile cancers worldwide are attributed to oncogenic HPV^11–13^.

On October 2009, the U.S. Food and Drug Administration licensed a prophylactic quadrivalent HPV vaccine named Gardasil (Merck & Co. Inc.) for males aged between 9 and 26 years, previously licensed for females in 2006^14^, in an effort to reduce the incidence of genital warts. The FDA added prevention of anal cancer both in males and females as an indication for vaccine use in December 2010^15^. Several previous studies estimated that the vaccine protected against infection by HPVs 6, 11, 16, and 18 types that could cause over 90% of genital warts and 70% of cervical cancers^16,17^. An on-going study has shown the HPV vaccine is effective, safe, and well-tolerated among adolescent and young adult women^18,19^. Meanwhile, data from vaccination trials have shown reductions in persistent HPV infections and genital warts among vaccinated males^20,21^. As with females, HPV vaccination is likely to offer the greatest benefit when administered before the onset of sexual activity and HPV exposure. For MSM, a recent study showed that a national vaccination program could result in high uptake of vaccine, through sex health services^22^. Predictors of vaccine acceptability for males are similar to those that influence intention to vaccinate among females and include perceived susceptibility to HPV infection, sexual activity, perceived benefits of the vaccine, and perceived norms for vaccination^23^. Nowadays, more and more countries approve HPV vaccination for females. However, male HPV vaccination programs have been in debate because of the cost-effectiveness^24,25^. In nowadays China, HPV vaccine has not been approved to males yet. Little was known about the awareness of the virus and the acceptability of the vaccine in males especially in high-risk subgroups such as MSM.

Our study aimed to investigate the knowledge and attitude towards HPV infection and HPV vaccination among MSM from mainland China by means of a multi-centered cross-sectional study. The potential factors which might influence the acceptability of HPV vaccination were identified in the study population as well.

## Results

### Subject recruitment and characteristics

In total, 3154 individuals were interviewed and signed informed consents. After excluding 97 participants who lacked necessary information on sexual behaviors, 3057 (96.9%) eligible participants were included in data analysis. As shown in Table 1, major characteristics of the included participants were presented by study sites. The mean age of participants was 31.0 years old with a standard deviation (SD) of 8.5. The first homosexual experience of the participants occurred at mean age of 21.7 (SD = 5.6). The majority of participants (74.5%, 2277/3057) self-reported a homosexual orientation, and more than one third of them (39.1%, 1195/3057) had more than 10 homosexual partners in their lifetime. More than two thirds of participants (76.2%, 2329/3057) were unmarried. There were 255 (8.3%) participants reporting a history of genital wart.

**Table 1:** Descriptive statistics for demographics and behaviors of the study population.

Variable	All sites n (%)	Developed Cities^a^ n (%)	Medium developed Cities^b^ n (%)	Less developed Cities^c^ n (%)	*P* for χ^2^ test
**(Part 1/2)**					
Total	3057	1202	1222	633	
Age (years, mean ± SD)	31.0 ± 8.5	30.7 ± 7.6	30.8 ± 9.0	32.2 ± 8.9	<0.01
Education					
≤9 years	328 (10.7)	96 (8.0)	154 (12.6)	78 (12.3)	<0.01
10–12 years	731 (23.9)	317 (26.4)	255 (20.9)	159 (25.1)	
>12 years	1998 (65.4)	789 (65.6)	813 (66.5)	396 (62.6)	
Marriage status					
Unmarried	2329 (76.2)	982 (81.7)	947 (77.5)	400 (63.2)	<0.01
Married	573 (18.7)	183 (15.2)	208 (17.0)	182 (28.7)	
Divorced or widowed	155 (5.1)	37 (3.1)	67 (5.5)	51 (8.1)	
Employment					
Own a business	337 (11.0)	97 (8.1)	153 (12.5)	87 (13.7)	<0.01
Employee	1732 (56.7)	789 (65.6)	579 (47.4)	364 (57.5)	
Freelance	320 (10.5)	99 (8.2)	151 (12.4)	70 (11.1)	
Not employed	668 (21.8)	217 (18.1)	339 (27.7)	112 (17.7)	
Self-reported sexual orientation					
Homosexual	2277 (74.5)	930 (77.4)	906 (74.1)	441 (69.7)	<0.01
Heterosexual	34 (1.1)	12 (1.0)	16 (1.3)	6 (0.9)	
Bisexual	615 (20.1)	213 (17.7)	247 (20.2)	155 (24.5)	
Not sure	131 (4.3)	47 (3.9)	53 (4.4)	31 (4.9)	
**(Part 2/2)**					
Ever had sex with women					
Yes	1116 (36.5)	334 (27.8)	461 (37.7)	321 (50.7)	<0.01
No	1941 (63.5)	868 (72.2)	761 (62.3)	312 (49.3)	
Age at first homosexual behavior (years, mean ± SD)	21.7 ± 5.6	21.7 ± 4.8	21.3 ± 5.7	22.3 ± 6.6	<0.01
Number of lifetime homosexual partners					
1–5	1004 (32.8)	353 (29.4)	470 (38.4)	181 (28.6)	<0.01
6–10	858 (28.1)	347 (28.9)	363 (29.7)	148 (23.4)	
11–30	718 (23.5)	266 (22.1)	277 (22.7)	175 (27.6)	
More than 30	477 (15.6)	236 (19.6)	112 (9.2)	129 (20.4)	
Have fixed sexual partner currently	1446 (47.3)	602 (50.1)	552 (45.2)	292 (46.1)	0.04
Ever been diagnosed with genital wart	255 (8.3)	88 (7.3)	99 (8.1)	68 (10.7)	<0.01
Ever been diagnosed with STIs except genital wart	478 (15.6)	249 (20.7)	141 (11.5)	88 (13.9)	<0.01

Abbreviation: SD, standard deviation; STIs, sexually transmitted infections; ^a^Developed Cities: Beijing, Shanghai, Guangzhou, Tianjin; ^b^Medium developed Cities: Xi’an, Chengdu, Chongqing, Wuhan; ^c^Less developed Cities: Zhengzhou, Taiyuan.

Data source: a multicenter cross-sectional study that conducted in men who have sex with men from 10 cities in mainland China between December 2012 and July 2014.

### Awareness of HPV, genital wart and anal cancer

Data regarding knowledge of HPV are presented in Table 2. Among the 3057 participants included in the study, 629 (20.6%) had heard about HPV. Meanwhile, 43.7% (1335/3057) and 76.1% (2326/3057) of all participants had never heard of genital wart and anal cancer, respectively. Approximately 29.3% (505/1722) and 49.1% (359/731) of the subjects had never worried about getting genital wart and anal cancer, respectively. No significant differences in awareness of genital wart and anal cancer were observed between the study participants grouped by local economic levels.

**Table 2:** Awareness of genital wart and anal cancer.

Item	All site n (%)	Developed Cities^a^ n (%)	Medium developed Cities^b^ n (%)	Less developed Cities^c^ n (%)	*p*-value
**(Part 1/2)**					
Genital wart awareness					<0.01
Never heard	1335 (43.7)	470 (39.1)	611 (50.0)	254 (40.1)	
A little	1161 (38.0)	533 (44.3)	397 (32.5)	231 (36.5)	
A moderate amount	422 (13.8)	157 (13.1)	157 (12.9)	108 (17.1)	
Quite a lot	139 (4.5)	42 (3.5)	57 (4.6)	40 (6.3)	
Do you worry about getting genital warts					<0.01
Not at all	505 (29.3)	147 (20.1)	246 (40.3)	112 (29.5)	
A little	700 (40.7)	318 (43.5)	246 (40.3)	136 (35.9)	
A moderate amount	322 (18.7)	178 (24.4)	74 (12.1)	70 (18.5)	
Quite a lot	194 (11.3)	88 (12.1)	45 (7.3)	61 (16.1)	
Anal cancer awareness					<0.01
Never heard	2326 (76.1)	857 (71.3)	987 (80.8)	482 (76.1)	
A little	575 (18.8)	277 (23.1)	183 (15.0)	115 (18.2)	
A moderate amount	130 (4.2)	58 (4.8)	41 (3.3)	31 (4.9)	
Quite a lot	26 (0.9)	10 (0.8)	11 (0.9)	5 (0.8)	
Do you worry about getting anal cancer					<0.01
Not at all	359 (49.1)	145 (42.0)	147 (62.5)	67 (44.4)	
A little	240 (32.8)	133 (38.6)	69 (29.4)	38 (25.2)	
A moderate amount	73 (10.0)	37 (10.7)	15 (6.4)	21 (13.9)	
Quite a lot	59 (8.1)	30 (8.7)	4 (1.7)	25 (16.5)	
Have you heard of HPV					
Yes	629 (20.6)	265 (22.1)	225 (18.4)	139 (22.0)	0.05
No	2428 (79.4)	937 (77.9)	997 (81.6)	494 (78.0)	
**(Part 2/2)**					
Can HPV be transmitted by sexual behavior					0.39
Yes	517 (82.3)	189 (84.0)	210 (79.3)	118 (85.5)	
No	21 (3.4)	5 (2.2)	11 (4.1)	5 (3.6)	
Not sure	90 (14.3)	31 (13.8)	44 (16.6)	15 (10.9)	
Can HPV cause genital warts					0.24
Yes	432 (68.9)	179 (67.6)	160 (71.4)	93 (67.4)	
No	29 (4.6)	8 (3.0)	11 (4.9)	10 (7.2)	
Not sure	166 (26.5)	78 (29.4)	53 (23.7)	35 (25.4)	
Can HPV cause anal cancer					0.02
Yes	345 (55.0)	139 (52.5)	130 (57.8)	76 (55.5)	
No	39 (6.2)	11 (4.1)	12 (5.3)	16 (11.7)	
Not sure	243 (38.8)	115 (43.4)	83 (36.9)	45 (32.8)	

Abbreviation: HPV, human papillomavirus; ^a^Developed Cities: Beijing, Shanghai, Guangzhou, Tianjin; ^b^Medium developed Cities: Xi’an, Chengdu, Chongqing, Wuhan; ^c^Less developed Cities: Zhengzhou, Taiyuan.

Data source: a multicenter cross-sectional study that conducted in men who have sex with men from 10 cities in mainland China between December 2012 and July 2014.

### Awareness and knowledge of HPV vaccine

**Figure 1:**
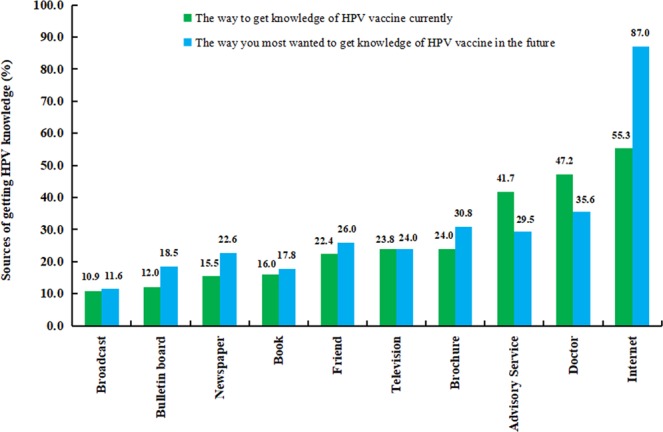
Percentage of HPV knowledge sources. The major sources of HPV vaccination information that the individuals most wanted to get in the future were internet (87.0%, 127/146), doctors (35.6%, 52/146), and advisory sevice (29.5%, 43/146).

Table 3 shows the knowledge on HPV vaccine among the participants before our knowledge lecture on HPV. Only 4.8% (146/3057) participants stated that they had heard of HPV vaccine. As shown in Fig. 1, the major sources of HPV vaccination information that the individuals most wanted to get in the future were internet (87.0%, 127/146), doctors (35.6%, 52/146), and advisory sevice (29.5%, 43/146). At post-test, the percentage of participants who were willing to get vaccination was 67.5% (Supplementary Table S1).

**Table 3:** Opinion on HPV vaccine among those with self-reported HPV vaccine awareness.

Item	All sites n (%)	Developed Cities^a^ n (%)	Medium developed Cities^b^ n (%)	Less developed Cities^c^ n (%)	*p*-value
Having HPV vaccine awareness	146 (4.8)	61 (5.1)	44 (3.6)	41 (6.5)	0.02
Ever thought about getting HPV vaccine					0.28
Yes	72 (49.3)	26 (42.6)	22 (50.0)	24 (58.5)	
No	74 (50.7)	35 (57.4)	22 (50.0)	17 (41.5)	
Opinion on the effect of HPV vaccine in general					0.14
Positive	80 (54.8)	26 (42.6)	27 (61.4)	27 (65.8)	
Neutral	59 (40.4)	32 (52.5)	15 (34.1)	12 (29.3)	
Negative	7 (4.8)	3 (4.9)	2 (4.5)	2 (4.9)	
Opinion on the effect of HPV vaccine for males					0.08
Yes	106 (72.6)	37 (84.1)	37 (84.1)	32 (78.0)	
No	6 (4.1)	3 (2.3)	1 (2.3)	2 (4.9)	
Refused to answer	34 (23.3)	21 (13.6)	6 (13.6)	7 (17.1)	

Abbreviation: HPV, human papillomavirus; Data source: a multicenter cross-sectional study that conducted in men who have sex with men from 10 cities in mainland China between December 2012 and July 2014.

### Attitudes toward HPV prevention and vaccination

Attitudes to the vaccination are shown in Table 4. The factors that might influence the willingness to accept HPV vaccination were safety of the vaccine (54.2%, 1656/3057) and severity of the infection (52.3%, 1599/3057). Moreover, the reasons that might promote willingness to be vaccinated include more attention to their health (75.1%, 2296/3057) and confidence to the vaccine efficacy (44.1%, 1347/3057). The places where they would like to get the vaccination include general hospitals (33.7%, 1030/3057), local Centers for Disease Control (32.4%, 990/3057), gay health centers (17.6%, 538/3057), and sexually transmitted disease clinics (10.1%, 309/3057).

**Table 4:** Attitudes toward HPV prevention and vaccination.

Item	All sites n (%)
**The factors might be barriers on HPV vaccination**	
Safety of HPV vaccine	1656 (54.2)
Severity of HPV infection	1599 (52.3)
Cost of HPV vaccination	767 (25.1)
Effectiveness of HPV vaccine	642 (21.0)
Hassle of HPV vaccination	198 (6.5)
Perceived likelihood of HPV infection	189 (6.2)
Maybe discriminated	142 (4.7)
**The factors might be facilitators on HPV vaccination**	
Pay more attention to my health	2296 (75.1)
Could effectively avoid from infecting HPV	1347 (44.1)
The HPV vaccine could prevent genital warts	1186 (38.8)
The vaccine could prevent cancer	1134 (37.1)
The vaccines are effective and security in most cases	716 (23.4)
The advantages outweigh the disadvantages	709 (23.2)
It might do good to others if I take it	563 (18.4)
**The place you might be most willing to be vaccinated**	
General hospitals	1030 (33.7)
Centers for Disease Control	990 (32.4)
Gay health centers	538 (17.6)
Sexually transmitted disease clinics	309 (10.1)
Community hospitals	155 (5.1)
Others	33 (1.1)

Abbreviation: HPV, human papillomavirus; Data source: a multicenter cross-sectional study that conducted in men who have sex with men from 10 cities in mainland China between December 2012 and July 2014.

**Figure 2:**
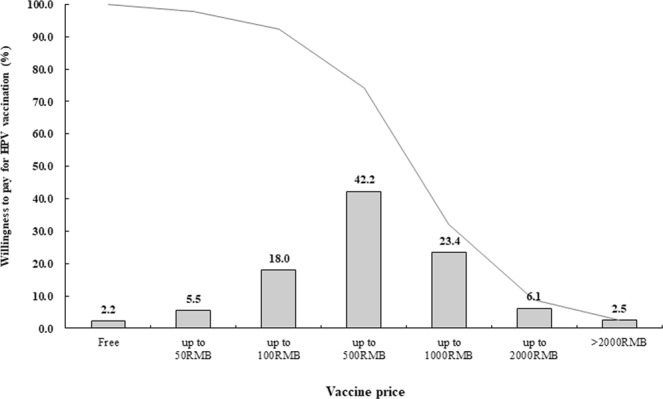
Willingness to pay for HPV vaccination. Bars represent the interval within which the maximum price willingness-to-pay being located, and solid line represents the cumulative proportion of participants whose willingness-to-pay for the price in the certain intervals. Data source: a multicenter cross-sectional study that conducted in men who have sex with men from 10 cities in mainland China between December 2012 and July 2014.

As shown in Fig. 2, a very small fraction (2.2%, 64/2946) of the participants stated that they would like to be vaccinated when the vaccine were provided for free. Among those who were willing to pay for vaccination, a J-shaped distribution of maximum willingness-to-pay was observed where 42.2%(1244/2946) of participants expressed their willingness-to-pay below 500 RMB, declining to 2.5% when price was higher than 2000 RMB (Fig. 2).

Supplementary Table S2 shows the proportional odds ordinal regression results with likeliness of vaccinating as the outcome variable. Due to relatively lower percentage, categories “very unlikely”, “unlikely” and “not sure” for vaccine acceptability were collapsed into one single category. A total of 3057 observations were included in the final model. Our results demonstrated that ever diagnosed with genital warts or other STDs, and heard of HPV or the vaccine before on vaccination practice were most strongly associated with stronger support for vaccination. However, no significant relation was found between vaccine acceptability and social-economic status which was represented by education level and monthly income.

## Discussion

To our knowledge, this is the first multicenter study from mainland China with a large sample size to investigate HPV awareness and vaccine acceptability among MSM. In total, 3057 eligible participants from 10 cities were investigated, only 20.6% of them had heard of HPV and 4.8% had heard of HPV vaccine. Factors that potentially influence willingness for HPV vaccination were found to be vaccine safety (54.2%) and severity of HPV infection (52.3%). Among those who would like to pay for HPV vaccination, 42.2% of participants would pay up to 500 RMB and only a minority of participants (2.5%) would afford more than 2000RMB.

The general knowledge on HPV was not satisfying in our study population. Only 20.6% of them have heard of HPV, it is significantly lower than the reports from western countries (73.6–93.0%)^26–28^. Nearly a half (43.7%) of the participants stated that they had never heard of genital warts, and much smaller number of them knew the correlation between HPV and genital warts and cancers. Lacking knowledge on HPV infection and related diseases will be a big challenge for the prevention and early diagnosis.

This study revealed that 36.5% of the participants ever had sex with women. It was close to similar studies from China, with a proportion between 27% and 56%, but it was higher than a similar study in the USA, which showed 20% of the participants ever had sex with female partners^5,29–32^. Apart from 20.1% of the study participants were self-reported bisexuality, another explanation could not be excluded. As only heterosexual marriage is currently legally allowed in China, approximately one fourth (23.6%) of the MSM population ever married due to pressure from family and society.

In the present study, correlates of HPV vaccine acceptability were generally consistent with those identified in several previous studies^33,34^. The men who paid more attention to their own health and who believed that HPV vaccine could effectively protect from infection had stronger willingness to get vaccinated. The participants who had been diagnosed with genital warts or other STD showed higher HPV vaccine acceptability, which indicated that men who were sexually active men and had practice for STD diagnosis and treatment might more care about disease prevention. It is important to note that although knowledge of HPV and the vaccine was quite poor in the study population, their attitudes toward vaccination and intentions to be vaccinated may change from moderate to favorable after announcement and education. Therefore, MSM in China, as a high-risk population, need more knowledge of HPV and related diseases, so that they can better understand the benefits of HPV vaccine. And it is an important premise for active self-protection by behavior change and by available tools including HPV vaccine.

In this study, HPV vaccine acceptability was uniquely predicted by several factors, including perceived benefits of the vaccine and severity of HPV infection to receive the HPV vaccine. It indicates that future HPV vaccination programs targeting at MSM should strengthen the health education on the risks of HPV infection and the benefits of vaccination. As we all know, current HPV vaccination campaigns typically highlight the benefits of the HPV vaccine for women. To some degree, it might be difficult to let all men be willing to receive a vaccine presented as a means of cervical cancer prevention. It must underscore the need for continued efforts aiming at removing barriers associated with HPV vaccination. In addition, high expense was found to be associated with diminished vaccine interest. What’s more, HPV screening is not an established clinical practice for men even for those with STD in China currently. All these difficulties to generalize HPV vaccination in high-risk male populations should be considered by public healthcare providers.

When interpreting the results of the study, some limitations should be kept in mind. First, socio-demographic and risk behaviors of participants were collected by the questionnaires. Therefore, potential bias caused by inaccurate responses cannot be excluded. Second, because the information on HPV and HPV vaccine was given by the trained investigators before asking for the acceptability of HPV vaccination, we could not exclude the possibility that the results might be influenced by the training efficacy. Third, due to the potential limitation of enrollment methods, our study participants might not represent the general MSM population from the study sites. Therefore, selection bias can not be excluded. Fourth, during the past decade, infection with high-risk HPVs, in particular HPV 16, has been newly recognized as risk factor for head and neck cancers, specifically arising in the oropharynx^35^. Our study lacks the investigation and education on this important issue among the study population. Fifth, cross-sectional study itself has some limitations on association analysis. Despite the limitations above, our study has important strengths. We examine acceptability of HPV vaccination using a large-scale sample of participants and the response rate was high.

In conclusion, our investigation showed that awareness and knowledge on HPV and the vaccine were quite poor among MSM in mainland China. Most of the study participants cared more about the safety and efficacy of the vaccine, and showed higher willingness for HPV vaccination after education. Therefore, we need input much more public health resources to generalize knowledge of HPV and related diseases in the male populations especially in the subgroups under high risks of STD, such as MSM. Clinic trails evaluating HPV vaccine application in males should be urged to accelerate the approval of HPV vaccination in needed males in China.

## Materials and Methods

### Ethics statement

The present study was approved by the Ethics Committees of the Institute of Pathogen Biology, Chinese Academy of Medical Sciences (ref IPB-2011-1). Written informed consent was obtained from the eligible study participants before interview. All methods were carried out according to the approved guidelines set by the Institute of Pathogen Biology, Chinese Academy of Medical Sciences & Peking Union Medical College.

### Study design

The present study was a cross-sectional study conducted in MSMs from 10 selected cities in mainland China (Beijing, Shanghai, Guangzhou, Tianjin, Xi’an, Chengdu, Chongqing, Wuhan, Zhengzhou, and Taiyuan). This study was coordinated by Institute of Pathogen Biology of Chinese Academy of Medical Sciences (Beijing, China). Local non-government organizations involved in the enrollment of the study participants: Beijing Rainbow Volunteers Workstation, Chengdu Yongle Volunteers Workstation, Chongqing Tongxin Volunteers Workstation, Wuhan Volunteers Workstation, Guangzhou MSM network station, Tianjin Deep Blue Volunteers Workgroup, Shanghai guanggong Volunteers Workstation, Taiyuan kangtong Volunteers Workstation, Xi’an Tongkang Volunteers Workstation and Zhengzhou Sanhe Volunteers Workstation.

According to the average income, the ten study sites were divided into three groups, including developed Cities (Beijing, Shanghai, Guangzhou, Tianjin), medium developed Cities (Xi’an, Chengdu, Chongqing, Wuhan) and less developed Cities (Zhengzhou, Taiyuan) by socioeconomics level from national bureau of statistics of the People’s Republic of China^36^.

### Study population

Study participants were recruited through local non-government organizations above from the selected cities between December 2012 and July 2014. Multiple methods were used for recruitment including website advertisement, distributing flyers with study-related information at MSM-frequented venues (e.g., MSM clubs, bars, parks and bathhouses), and eligible study participants were also encouraged to refer their peers to attend the study. Meanwhile, we encouraged eligible study participants to introduce their peers to attend the study. The inclusion criteria were males, ≥18 years old, ever having homosexual behaviors, having not been given HPV vaccine and willing to provide written informed consent.

### Data collection

Trained interviewers collected sociodemographic information and self-reported health status by using a standardized questionnaire and administered in a private room. We collected sociodemographic information (including age, ethnicity, education record, monthly income, employment, and marriage status), sexual behavior, knowledge on HPV and HPV vaccine, and the acceptability of the HPV vaccine. Meanwhile, we also collected HPV awareness (whether they had heard of HPV) and the ways they heard about HPV. Moreover, we assessed HPV knowledge in the baseline and the post-test survey.

### Statistical analysis

Questionnaires were entered twice independently using EpiData software (EpiData Version 3.1, EpiData Association Odense, Denmark). After cleaning, we analyzed the data by Statistical Analysis System (SAS 9.12 for Windows; SAS Institute Inc., NC, USA). All of the included participants were characterized by living site with respect to age, marriage status, ethnicity, education record, age at the first homosexual act, ever having sex with women, self-reported sexual orientation, and whether anal sex a regular sex behavior. Differences between sites in these variables were compared using Pearson’s chi-square test. Changes of HPV knowledge from baseline to post-test by condition were assessed by a mixed design analysis of variance. To estimate the willingness-to-pay of the HPV vaccine, we excluded those who refused to take vaccinate and those who would vaccinate only when the vaccine is provided for free, assuming that lower bound of willingness-to-pay cannot be less than zero. Then an interval regression model was fit to the intervals of the true willingness-to-pay using Proc Lifereg procedure in SAS 9.12.

## Supplementary information

Human Papillomavirus awareness and vaccine acceptability among men who have sex with men from mainland China

## Data Availability

All data generated or analysed during this study are included in this published article (and its Supplementary Information Files).
