# Supplementary material for: Human Papillomavirus awareness and vaccine acceptability among men who have sex with men from mainland China
Source: Sci Rep. 2019 Jun 19;9:8763. doi: 10.1038/s41598-019-45258-0 (PMC6584641; doi:10.1038/s41598-019-45258-0)
Supplement: Supplementary file 1 — Human Papillomavirus awareness and vaccine acceptability among men who have sex with men from mainland China [file 41598_2019_45258_MOESM1_ESM.pdf]

Human Papillomavirus awareness and vaccine acceptability among men who have sex with men from mainland China

Xiangwei Li[1*], Xuefang Cao[1*], Zhen Li[2], Yu Yang[3], Mufei Li[1], Boxuan Feng[1], Henan Xin[1], Haoran Zhang[1], Lei Gao[1]†

[1]NHC Key Laboratory of Systems Biology of Pathogens, Institute of Pathogen Biology, Chinese Academy of Medical Sciences & Peking Union Medical College, Beijing, China

[2]Chaoyang Centers for Disease Control and Prevention, Beijing, China

[3]Department of Epidemiology and Biostatistics, School of Public Health, Peking University Health Science Centre, Beijing, China

*These two authors contributed equally.

†**Correspondence:** Prof. Lei Gao, Institute of Pathogen Biology, CAMS & PUMC. Dong Dan San Tiao 9.100730 Beijing, China. Phone: 86-10-67828550. Fax: 86-10-67828550. * Email: gaolei@ipbcams.ac.cn

Supplementary Table S1. HPV vaccine knowledge among the study population after training

Question	Strongly disagree	Disagree	No opinion	Agree	Strongly agree
Vaccination willingness	67(2.2)	160(5.2)	767(25.1)	897(29.4)	1164(38.1)
HPV vaccine can prevent genital warts	107(3.5)	330(10.8)	1036(33.9)	1315(43.0)	267(8.7)
HPV vaccine can prevent anal cancer	109(3.6)	349(11.4)	1142(37.4)	1203(39.4)	252(8.3)
Might be diagnosed anal cancer in future if you are not vaccinated with HPV vaccine	135(4.4)	1233(40.4)	1109(36.3)	514(16.8)	64(2.1)
Might be diagnosed genital warts in future if you are not vaccinated with HPV vaccine	188(6.2)	1250(40.9)	1127(36.9)	447(14.6)	43(1.4)
After vaccination with HPV vaccine, you will not get genital warts	247(8.1)	2016(66.0)	674(22.1)	98(3.2)	19(0.6)
After vaccination with HPV vaccine, you will not get anal cancer	266(8.7)	2033(66.6)	641(21.0)	97(3.2)	15(0.5)

Supplementary Table S2. Proportional odds ordinal regression results associated with HPV vaccine acceptance

Independent variables	n	Very likely	Likely	Not sure	Unlikely	OR(95% CI)	*p*-value
Age (mean±SD)	3057 (31.0±8.5)	1163 (31.5±8.5)	895 (31.1±8.6)	772 (30.3±7.7)	227 (30.4±8.5)	0.9 (0.9,1.0)	<0.01
Education							
≤9 years	328	148(12.7)	91(10.2)	64(8.3)	25(11.0)	Ref.	/
10–12 years	731	265(22.8)	207(23.1)	193(25.0)	66(29.1)	0.7(0.6,1.0)	0.02
＞12 years	1998	750(64.5)	597(66.7)	515(66.7)	136(59.9)	0.9(0.7,1.0)	0.08
Monthly income							
No Income	525	231(19.9)	163(18.2)	96(12.4)	35(15.4)	Ref.	/
<1000RMB	94	39(3.4)	31(3.5)	20(2.6)	4(1.8)	0.6(0.5,0.7)	<0.01
1000-3000RMB	1176	461(39.6)	327(36.5)	286(37.1)	102(44.9)	0.8(0.5,1.2)	0.30
3000-5000RMB	847	267(23.0)	276(30.8)	246(31.9)	58(25.6)	0.9(0.7,1.1)	0.23
>5000RMB	414	165(14.2)	98(11.0)	124(16.1)	28(12.3)	1.1(0.9,1.3)	0.55
Ever diagnosed with genital warts							
No	2802	1025(88.1)	830(92.7)	734(95.1)	213(93.8)	Ref.	/
Yes	255	138(11.9)	65(7.3)	38(4.9)	14(6.2)	1.5(1.2,2.0)	<0.01
Ever diagnosed with STI except genital warts							
No	2579	936(80.5)	756(84.5)	687(89.0)	200(88.1)	Ref.	/
Yes	478	227(19.5)	139(15.5)	85(11.0)	27(11.9)	2.1(1.7,2.5)	<0.01
HPV awareness							
Never heard of the HPV before	2428	821(70.6)	724(80.9)	685(88.7)	198(87.2)	Ref.	/
Heard of the HPV before	629	342(29.4)	171(19.1)	87(11.3)	29(12.8)	1.3(1.1,1.6)	0.01
HPV vaccine awareness							
Never heard of the HPV vaccine before	2627	949(81.6)	779(87.0)	702(90.9)	197(86.8)	Ref.	/
Heard of the HPV vaccine before	430	214(18.4)	116(13.0)	70(9.1)	30(13.2)	1.2(1.0,1.5)	0.08

Abbreviation: CI, confidence interval; HPV, human papillomavirus; OR, odd ratio; SD, standard deviation; STIs, sexually transmitted infections;

Data source: a multicenter cross-sectional study that conducted in men who have sex with men from 10 cities in mainland China between December 2012 and July 2014.
